# Implantable Cardioverter-defibrillator Use in Structural Heart Disease: Narrowing the Playing Field

**DOI:** 10.19102/icrm.2018.090207

**Published:** 2018-02-15

**Authors:** Christopher R. Ellis

**Affiliations:** ^1^Vanderbilt Heart and Vascular Institute, Nashville, TN, USA

**Keywords:** Defibrillation, sudden death, ventricular arrhythmias

The manuscript in this issue of *The Journal of Innovations in Cardiac Rhythm Management* by Boey et al.^1^ reviews sudden cardiac death (SCD) risk prevention for patients with structural heart disease and impaired left ventricular function. Patients with inherited channelopathies (eg, long QT syndrome, short QT syndrome, catecholaminergic polymorphic ventricular tachycardia); genetic disorders such as arrhythmogenic right ventricular dysplasia (ARVD) or hypertrophic cardiomyopathy (HCM); and those with unique statuses such as having cardiac sarcoidosis or awaiting heart transplantation are not reviewed. Unfortunately, the lack of prospective randomized trials for these conditions means they will likely never reach class I or IIa indications for implantable cardioverter-defibrillator (ICD) implantation, despite the obvious potential benefit to patient survival.

Prospective randomized clinical trials for ICD implantation for secondary prevention have firmly established the role of the ICD in preventing recurrent sudden death. Primary prevention ICD trials involving high-risk subjects post-myocardial infarction who have a left ventricular ejection fraction (LVEF) < 30% for more than 40 days or an ischemic LVEF < 35% and class II/III systolic heart failure are similarly strongly supported. As the authors nicely review, the benefit of primary prevention ICD implantation in the nonischemic cardiomyopathy patient has been questioned. The DANISH ICD trial^2^ raises important questions and perhaps the largest benefit for nonischemic cardiomyopathy subjects with left bundle branch block and QRS duration > 120 ms is from cardiac resynchronization therapy (CRT) pacing rather than the presence of a defibrillation lead. A randomized trial comparing the use of CRT-pacemakers and CRT-defibrillators would be greatly welcome in this population, in particular in elderly patients.

Early implantation of an ICD has not proven effective at reducing overall mortality (DINAMIT trial^3^) and supports the expanding consideration of covering the high-risk patient with a LifeVest (Zoll Medical, Inc., Chelmsford, MA, USA) or a “wearable defibrillator.” Deferring the decision to implant a permanent ICD until medical therapy has been optimized and the subject has proved the ability to survive the early at-risk period is an evolving option needing further study. The majority of LifeVest (Zoll Medical, Inc., Chelmsford, MA, USA) use at Vanderbilt Medical Center is post-lead extraction in patients awaiting reimplantation. However, the rapid growth of our ventricular assist device and heart transplant program has expanded routine LifeVest (Zoll Medical, Inc., Chelmsford, MA, USA) use to early on while awaiting heart transplant evaluation in some patients, avoiding the risk of invasive ICD implantation altogether.

Although the use of the subcutaneous ICD (S-ICD) may reduce the potential risks of lead dislodgement, pneumothorax, and fracture, these devices are often implanted in the worst clinical candidates. Patients with prior cardiac implantable electronic device infections have a high one-year mortality and those with end-stage renal disease have not demonstrated any benefit from ICD therapy. Importantly, these are often the patients S-ICD implantation is first considered in, thus limiting the potential widespread benefit of the therapy. Additionally, implantations are typically performed under general anesthesia, adding some cognitive risk. S-ICDs can be prone to undersensing when liberties are taken with screening electrocardiograms to determine adequate QRS and T-wave morphology in order to quality a patient for device implantation, or when they are implanted in obese subjects. An unintended benefit of the delayed S-ICD detection and charge time to reach 65 Joules (J) or 80 J is the allowance for more sustained ventricular arrhythmias to prove themselves to be “non-sustained” by spontaneously converting prior to ICD shock. Though the youngest patients may derive the greatest long-term benefit from S-ICD implantation, this is also generally the population of patients with hypertrophic cardiomyopathy, long QT syndrome, or ARVD who are prone to inappropriate sensing and/or T-wave double-counting and who often benefit from the presence of antitachycardia or atrial pacing support.

Finally, when considering the use of an electrophysiology study (EPS) to induce ventricular tachycardia (VT) or ventricular fibrillation (VF) for the placement of a primary prevention ICD in patients with an LVEF > 35%, the devil is in the details. There is no specific, uniform induction sequence for the EPS and, depending on the operator’s desire to implant a device, the protocol can be tailored to either increase or decrease the likelihood of inducing VT, VF or ventricular flutter. VF is not primarily a reentrant arrhythmia and, with the progress and efficacy of VT ablation, patients with induced VT in the electrophysiology laboratory may be better suited for VT ablation and implantable loop recorder monitoring for spontaneous events. This would be fertile ground as well for a randomized trial in the future on the tail of the SMASH-VT^4^ and VTACH^5^ trials.
